# The Application of Multi-Parameter Multi-Modal Technology Integrating Biological Sensors and Artificial Intelligence in the Rapid Detection of Food Contaminants

**DOI:** 10.3390/foods13121936

**Published:** 2024-06-19

**Authors:** Longlong Zhang, Qiuping Yang, Zhiyuan Zhu

**Affiliations:** 1 Key Laboratory of Intelligent Manufacturing Technology (Shantou University), Ministry of Education, Shantou 515063, China; 2 College of Electronic Engineering, Southwest University, Chongqing 400715, China; 3 Hubei Key Laboratory of Food Nutrition and Safety, Huazhong University of Science and Technology, Wuhan 430030, China

**Keywords:** artificial intelligence, biosensors, feature extraction, machine vision, data analysis

## Abstract

Against the backdrop of continuous socio-economic development, there is a growing concern among people about food quality and safety. Individuals are increasingly realizing the critical importance of healthy eating for bodily health; hence the continuous rise in demand for detecting food pollution. Simultaneously, the rapid expansion of global food trade has made people’s pursuit of high-quality food more urgent. However, traditional methods of food analysis have certain limitations, mainly manifested in the high degree of reliance on personal subjective judgment for assessing food quality. In this context, the emergence of artificial intelligence and biosensors has provided new possibilities for the evaluation of food quality. This paper proposes a comprehensive approach that involves aggregating data relevant to food quality indices and developing corresponding evaluation models to highlight the effectiveness and comprehensiveness of artificial intelligence and biosensors in food quality evaluation. The potential prospects and challenges of this method in the field of food safety are comprehensively discussed, aiming to provide valuable references for future research and practice.

## 1. Introduction

In the contemporary food industry, globalization and technological advancement are driving the increasing demand for food safety and quality among consumers [1]. This trend becomes particularly significant under rapid economic and social development, with a growing concern for individual health issues. Food safety, as an indispensable element in safeguarding human health, has garnered widespread attention, encompassing the physical properties, nutritional value, and preventive measures of food [2]. Meanwhile, the rapid growth in food trade has made high-quality food a key factor in market competition [3]. However, food is a complex entity composed of multiple components, making comprehensive quality and safety assessment crucial [4]. Currently, the applicability and completeness of food safety assessment have not been fully established, lacking corresponding risk assessment systems and technological frameworks [5]. Moreover, the assessment methods are cumbersome, time-consuming, and costly, with subjective biases and insufficient scientific bases, making large-scale food quality monitoring and assessment impractical [6]. Therefore, to ensure food quality and safety, continuous monitoring of product quality characteristics during transportation and storage is paramount. One method for food safety prevention is to utilize conventional technologies to detect food quality during transportation, such as chromatography [7], ultraviolet detection [8], or fluorescence techniques [9], combined with separation techniques [10]. However, these analytical methods have limitations. Since these analyses are performed post-production, they may lead to contaminated food already entering the market. Moreover, they are labor-intensive, intricate, and costly, demanding a substantial number of samples and proficient operators.

In this context, biosensors, as rapid, sensitive, and highly specific analytical devices, offer new possibilities for addressing food safety issues. Biological sensors in the food industry can be analogized to infrared photodetectors [11], audiovisual photodetectors [12], synergistic graphene photodetectors [13], potential fluctuation engineering-enhanced graphene photodetectors [14], and ultra-high-gain short-wave infrared detectors [15] used in the fields of communication and detection. These photodetectors enhance information acquisition and processing in communication and detection. Similarly, biological sensors in the food industry, through real-time monitoring and precise analysis, enhance food safety and quality control. Biosensors utilize biological receptors (such as enzymes, antibodies, etc.) combined with sensors to generate measurable signals through specific physicochemical transformations, thereby determining the molecular content in samples [16]. The selection of biosensors and sensors depends on the characteristics of the sample and the type of measurable characteristic considered. Biological receptors represent the key components of biosensors, responding only to specific analytes and not to other biological receptors present in the analytical sample. Compared to traditional chemical detection methods, biosensors offer advantages such as rapid response, ease of operation, and low cost, thus being widely applied in various stages of food production, processing, and sale. Through biosensors, real-time monitoring of food quality and safety is achievable, enabling prompt detection of issues and actions to ensure food safety and health.

However, due to the diversity and complexity of food safety issues, a single biosensor may not cover all potential food safety hazards, necessitating comprehensive assessment with other technologies and means [17]. In recent years, innovative technologies such as artificial intelligence have been introduced into the field of food safety, proposing various advanced food quality inspection strategies, including AI methods [18,19,20], red and purple spectroscopy, and biosensors [21,22,23,24,25]. Among them, the combination of artificial intelligence and biosensors has given rise to a new detection mode, which utilizes complex AI algorithms to transform information obtained by sensors, such as physical, chemical, biological, environmental, or identity-related parameters, into decision processes with enhanced accuracy and intelligence. Currently, AI biosensors have been widely applied in continuous glucose monitoring [26] and rapid pathogen detection [27], and they have made significant progress in the development of wearable electronic devices [28]. The continuous development and application of these technologies provide new avenues and possibilities for comprehensive monitoring and analysis of food quality and nutrition.

This paper aims to explore the latest advances in artificial intelligence and biosensors in acquiring external indicators and internal characteristics of food products. By comprehensively reviewing key research on convolutional neural networks and biosensors in the field of food quality perception and classification, this paper aims to highlight the synergistic role of artificial intelligence and biosensors in the perception and classification processes of food quality and to describe how biosensors and deep learning are combined to comprehensively assess food quality.

## 2. The Role of Biosensors in Food Safety Assessment

**Figure 1:**
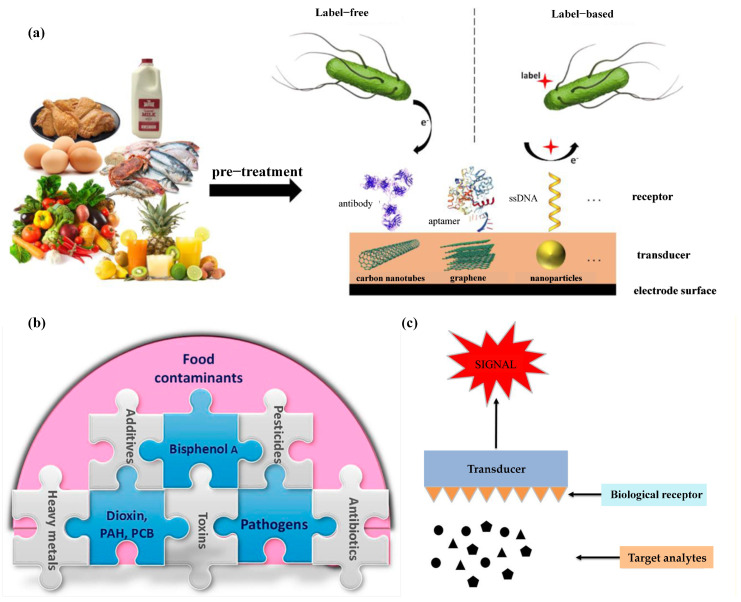
Application and evaluation of biological sensors in food. (**a**) Diagram illustrating biological sensor with close contact between sensor and receptor [29]. (**b**) Predominant food contaminants in food industries [38]. (**c**) Fundamentals of biosensors [40].

The role of biosensors in food safety assessment cannot be overlooked. The monitoring and analysis of contaminants in food are crucial steps to ensure food safety and public health. Jordi et al. [29] suggested that biosensors are analytical devices that can serve as an alternative to traditional methods for detecting pathogenic bacteria in food (Figure 1a). These sensors consist of biological components, such as antibodies, aptamers, or single-stranded DNA (ssDNA) receptors, that interact with target analytes (e.g., the pathogens depicted in the figure). The sensing layer, coupled with suitable transducers like carbon nanotubes, graphene, or nanoparticles, generates measurable signals from these biorecognition events. Various signal detection methods, including impedimetric, amperometric, and voltammetric techniques, allow biosensors to produce responses proportional to the concentration of the target analytes. This technology can be used for the pre-treatment and detection of pathogens in various foods. In label-free biosensors, the sensor directly generates a signal from the biorecognition event between the target analyte and the receptor. Conversely, label-based biosensors require a secondary molecule or aggregate (label) to generate a measurable signal, as the biorecognition event alone does not produce one. Figure 1 illustrates a typical label-based biosensor, where one receptor (e.g., an antibody or aptamer) is immobilized on the biosensor to capture the target analyte. Subsequently, another receptor (e.g., labeled ssDNA) binds to the captured analyte, and an appropriate molecular label produces the signal. These sensors convert biological reactions into assessable and convertible signals [30] and, with the support of modern technology, translate biological signals into electrical signals. Biosensors comprise molecular recognition and transduction components, converting biological signals into electrical signals through modern technology. Their structure includes a biological membrane and physical or chemical transducers used for the selective determination of analytes, thereby detecting viruses in food, water, environment, and agriculture [31]. By coating synthetic nanomaterials on biosensors, sensing electrodes can be modified to enhance sensing parameters, and signals can be conveyed to individuals through optical, piezoelectric, electrochemical, etc., means [32]. These signals are ultimately converted into electrical signals indicating the presence of biochemical targets, accessible via cloud-based data access. The outcomes of biosensors can be presented in graphical, tabular, or mathematical research formats [33].

The pioneering glucose oxidase (GOx) biosensor debuted in 1962, laying the foundation for biosensor technology [34]. Despite subsequent advancements since the 1960s, GOx sensors remain prevalent, representing the most favored type. These sensors offer advantages such as light weight, ease of use, portability, high detection limits, small analyte volumes required, and applicability in complex biological fluids. However, challenges they face include issues where surface topological structures fail to exhibit sufficient sensitivity and unique associations with target biologics, as well as challenges posed by pH values and ionic strength of biological analytes, leading to varied reactions [35]. These challenges can be addressed by integrating artificial intelligence (AI) technology with biosensors. Next-generation biosensor arrays incorporate AI algorithms, enabling them to exhibit higher specialization, selectivity, responsiveness, and consistency. These sensor arrays integrate solid-state and surface mechanical knowledge with integrated circuits, bioengineering, and data processing. With AI assistance, they can more accurately identify biological analytes, enhancing sensor performance and reliability [36]. Therefore, attention should now be directed towards the latest advancements in this field and outlining directions for the future development of AI-based biosensors.

In addition to enzyme biosensors [37], other types of biosensors such as optical and piezoelectric sensors have also been applied in the detection of toxins and chemicals in food production. Neethirajan et al. [38] illustrated the complexity of food contamination, highlighting common food contaminants and their interrelationships (Figure 1b). Different types of contaminants intertwine, making food safety monitoring complex and diverse. Safeguarding food safety requires comprehensive consideration and response to these different types of contaminants, taking effective measures to reduce their impact on human health and ensure the health and sustainability of the food supply chain [39]. Phumlani Tetyana et al. [40] proposed that the principle of each biosensor is based on molecular recognition, making its characterization crucial (Figure 1c). Effective characterization of biosensors requires comprehensive consideration of factors such as endogenous compounds and contaminants in food; therefore, the adoption of appropriate experimental methods and technological means is necessary. Amidst the prevailing challenges in food safety assessment, the integration of artificial intelligence (AI) with biosensors has assumed paramount importance [41]. Key attributes, including sensitivity, selectivity, stability, detection limit, reproducibility, response time, and range or linearity [42], are not only pivotal for the standalone performance of biosensors but also hold pivotal roles in their amalgamation with AI. This integration allows for the precise identification and interpretation of signals from biosensors, enabling the swift detection and accurate analysis of harmful substances in food [43]. AI algorithms heavily depend on the stable and consistent data furnished by sensors, highlighting the indispensability of sensor stability for ensuring algorithmic robustness and reliability. Low detection limits and high reproducibility enhance data quality, allowing AI models to predict and analyze the presence and concentration of harmful substances in food samples with greater accuracy [44]. The response time directly influences the real-time operational efficacy of AI systems, a crucial aspect for timely detection of food safety issues. Lastly, by optimizing range or linearity, AI models can adeptly adapt to data spanning varying concentration ranges, thereby augmenting the precision and credibility of food safety assessments. The amalgamation of these attributes is poised to propel technological innovation and advancement in the realm of food safety, furnishing indispensable support for public health.

## 3. Multi-Modal Model Based on Artificial Intelligence and Biosensors

**Figure 2:**
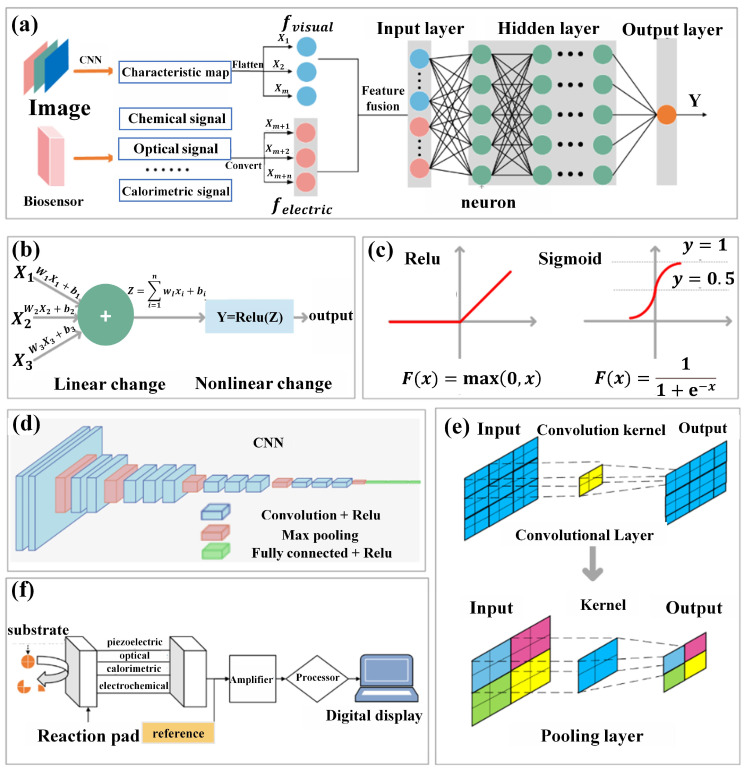
(**a**) Overall structure of the fusion model. (**b**) Structure and function of neurons. (**c**) Common nonlinear activation functions. (**d**) Overall structure of convolutional neural networks. (**e**) Convolution and pooling operations. (**f**) General composition of biosensors.

Integrating intelligent recognition methods that combine visual signals and bioelectric signals represents a pioneering direction in the field of food quality perception and classification [45]. This approach integrates computer vision with biosensor technology, aiming to overcome the inherent limitations of single-mode perception and human dependency in traditional methods [46]. At its core lies the fusion model structure [47], which comprises a feature extraction fusion stage and a perception scoring stage. As depicted in Figure 2a, in the feature extraction fusion stage, food image data capturing samples of varying qualities are generated and fed into a convolutional neural network (CNN) as input. Through multiple stacked convolutional and pooling layers, the CNN progressively extracts complex and abstract representations of input food images, forming one-dimensional feature vectors containing attributes such as color, texture, and overall appearance. Simultaneously, relevant substance information is transformed into sequential representations by biosensors. Then, feature vectors extracted from both image feature vectors and bioelectric signal sequences are concatenated as input to train the Deep Neural Network (DNN) model. Constructing the structured mathematical framework of the DNN model through extensive datasets, neural network weights and biases embedded within are trained. In the forward propagation algorithm, a series of linear operations are executed using multiple weight coefficient matrices and bias vectors, while activation functions facilitate the transformation from linear to nonlinear (Figure 2b). The selection of activation functions plays a crucial role in network learning and expressing nonlinear relationships, with commonly used activation functions including ReLU and Sigmoid functions (Figure 2c). Forward propagation transmits input data through hidden layers, ultimately generating the network’s output to achieve classification, regression, and other tasks. Upon completing forward propagation, the difference between predicted output and actual output is measured by a loss function, and weights and biases are updated using gradient descent methods to optimize the model parameters.

On the other hand, the primary objective of computer vision is to automate visual decision making by simulating human cognitive capabilities [48]. By learning from extensive datasets, algorithms in computer vision can make precise, efficient, and complex judgments [49]. A mature method within this field uses CNNs, which implement convolution through absorbing convolutional operations. The structure of CNNs, as depicted in Figure 2d, comprises input, convolution, pooling, and fully connected layers, among others, focusing on extracting information from input images. This information encompasses different aspects such as the texture and color of the images. Convolutional layers extract these attributes through convolution operations. This process entails traversing convolution filters over input images with defined strides, conducting cross-correlation computations to generate valuable feature maps [50]. Pooling layers play a crucial role in selecting the most significant features from convolutional layers. The overall configuration of convolution and pooling layers is illustrated in Figure 2e.

In the process of food evaluation and grading, there is no necessity to quantitatively acquire specific values of food attributes such as color and texture. Rather, reliance on neural networks for internal analysis is crucial [51]. Convolutional and pooling layers are fundamental building blocks in CNNs for extracting features from input images. Convolutional layers perform local feature extraction through convolution operations, capturing edges, textures, shapes, and other local features. Pooling layers reduce the spatial dimensions of feature maps through downsampling and aggregation operations, enhancing network efficiency and generalization. Multiple convolution kernels typically expand single feature maps into multiple smaller-sized feature maps, compensating for feature loss resulting from size reduction during convolution and pooling processes, essentially trading quantity for quality. Parallel learning and capturing of different types of features by multiple convolution kernels, parameter sharing to reduce the number of parameters, and sparse connections to reduce computational costs characterize the CNN. The translation invariance and feature compression properties of pooling layers make the network insensitive to positional changes, enhancing model robustness. By alternately using convolutional and pooling layers, CNN gradually extracts more abstract and advanced features, providing robust image analysis and recognition capabilities for subsequent food quality perception and grading tasks [52]. Currently, CNNs have been employed to address various challenges in the food domain, including food recognition, calorie estimation, foreign object detection, and maturity assessment [53]. However, leveraging computer vision techniques to solve challenges in the food domain inevitably relies on visual image features, which may have limitations in cases requiring maturity detection and quality assessment [54]. Relying solely on surface analysis of food appearance for detection and classification may result in incomplete evaluations. Therefore, there is an urgent need to explore a comprehensive approach that integrates internal and external factors to assess food properties more comprehensively.

As illustrated in Figure 2f, biosensors automatically analyze intrinsic food components through a series of systematic processes. Firstly, an automated sampling and preprocessing system ensures precise acquisition of food samples while minimizing potential variations due to human intervention. Subsequently, bioactive substances are selected based on the characteristics of target components to induce specific reactions that generate measurable signals. These signals are then detected and recorded using specialized sensor devices (such as optical, electrochemical, or bioluminescent sensors). Finally, advanced data analysis algorithms interpret and quantify these signals to determine the concentration of target components in the food matrix. By integrating the capabilities of automated sampling, carefully selected bioactive substances, signal detection, real-time monitoring, and remote control, biosensors achieve efficient, accurate, and streamlined analysis of intrinsic food components, reducing reliance on cumbersome manual procedures [55].

**Figure 3:**
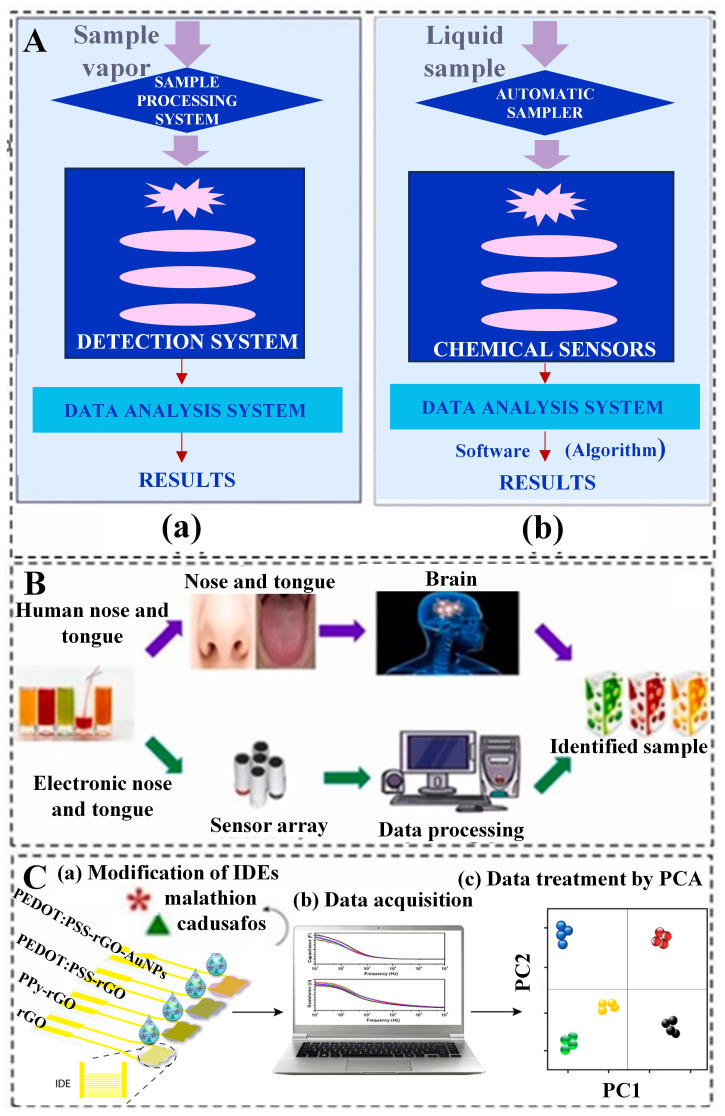
The application of artificial intelligence and biosensors in the food domain. (**A**) Schematic diagram of biosensor simulation (**a**) The simulation of the human olfactory system (**b**) The human gustatory system by biosensors [63] (reproduced from [63], with permission from Elsevier, 2016). (**B**) A comparison of the working principles of the gustatory system and the olfactory system [64] (reprinted (adapted) with permission from [64]. Copyright 2014 American Chemical Society). (**C**) A schematic diagram of an intelligent biosensor system based on graphene hybrid nanocomposites, used for detecting pesticide residues in agricultural products and statistical techniques for processing data collected by biosensors (reproduced (adapted) from [65], with permission from Elsevier, 2022) [65].

Building upon the principles of biosensors and artificial intelligence technology, researchers have developed various types of AI biosensors to detect specific substances. For instance, biosensors utilizing glucose [56], vitamins [57], tyramine [58], bisphenol A [59], and L-glutamate [60] have been developed to measure trace elements in food. Changes in the content of trace elements in food may be caused by various factors, such as nutrient-poor soil, adverse environmental conditions, or contamination during additive application or processing. The integration of artificial intelligence and biosensors brings significant advantages. Artificial intelligence can accelerate the process of data analysis, improving the sensitivity and accuracy of biosensors. By utilizing machine learning algorithms, artificial intelligence can identify complex patterns and trends, thereby enhancing the detection and analysis of trace element content [61]. Junjie, et al. [62] explained that biological sensors simulate the human olfactory system for rapid detection and identification of mixed odors. Miguel Peris et al. [63] proposed the use of specialized chemical sensors to mimic human odors, producing unique odor signatures when interacting with gas mixtures. These signatures are then compared with standard odor profiles to identify the mixed components. The biosensor system primarily consists of three parts (see Figure 3A(a)): (1) a sample processing system, (2) a detection system, and (3) a data processing system. This device has extensive applications in the quality assessment of agricultural products and food. The core of this system is a sensor array, where the excitation of sensor elements produces signals that generate unique responses to specific odor samples (odor fingerprints). This type of biosensor, made from biological materials, converts biological signals into electrical or optical signals. The biosensor, simulating the human taste system, comprises three parts: (i) an autosampler (optional), (ii) a set of chemically selective sensors, and (iii) signal processing algorithm software (see Figure 3A(b)). Biosensors emulate the human senses of smell and taste; electronic noses and tongues mimic this process by detecting samples through sensor arrays. Subsequently, a data processing system analyzes these data and identifies the samples, enabling high-sensitivity and accurate qualitative and quantitative determination of chemicals in complex samples. Magdalena Śliwińska et al. [64] introduced a sensor-simulated taste system consisting of three key components: a sample distribution chamber or optional automatic sample distributor, a sensor array with varying selectivities, and software for data processing designed to mimic the functionality of the brain’s image recognition systems. This innovative system enables the classification of chemical odors in liquid samples, as illustrated in Figure 3B. Facure et al. [65] prepared four types of nanocomposite thin-film sensor arrays by drop-casting solutions of rGO, PPy-rGO, PEDOT: PSS-rGO, and PEDOT: PSS-rGO-AuNPs onto gold interdigitated electrodes (IDEs). The sensing units were characterized by impedance spectroscopy and other methods, and the data were processed by Principal Component Analysis (PCA) (Figure 3C). Through the fine-tuning of design parameters of the sensors, especially for complex signals generated by sensor arrays, such as material selection, sensor structure, and detection methods, more reliable and continuous monitoring of trace elements can be achieved. This optimization not only significantly extends the lifespan of sensors but also improves their stability, making them more effectively applicable in practical food detection. Therefore, the combination of artificial intelligence and biosensors can provide a more comprehensive, accurate, and efficient solution for food safety assessment, aiding consumers in assessing the adequacy of their nutritional needs and assisting regulatory agencies in monitoring and managing food safety.

## 4. Discussion

The integration of biosensors and artificial intelligence (AI) offers several distinct advantages for systematically assessing food quality, including automation, efficiency enhancement, and cost-effectiveness [66]. However, several obstacles need to be addressed to make this approach feasible for food quality assessment. One key barrier involves establishing reliable and fully operational sensing systems capable of effectively detecting and analyzing various components present in different types of food. Additionally, careful consideration is required when selecting appropriate biosensors that align with the unique nutritional characteristics of different food categories [67]. Another significant issue revolves around relying on extensive statistical data to construct accurate assessment models, necessitating dedicated hardware resources. To address these challenges, advanced technologies such as chip integration, cloud computing, 5G networks, and other innovative solutions can be utilized effectively [68]. Combining biosensors with microchip integration has enabled the miniaturization and portability of sensing devices, facilitating real-time monitoring of on-site food quality [69]. Progress in cloud computing technology and big data analytics allows sensor data to be seamlessly uploaded to the cloud for efficient processing and storage. This enables rapid analysis of large-scale food samples, ensuring the maintenance of superior quality and safety standards. The advanced capabilities of 5G networks, including high-speed and low-latency attributes, establish robust and efficient communication infrastructure, enhancing the reliability and timely dissemination of sensor data for real-time monitoring purposes [70]. In addition to the aforementioned factors, integrating other complementary solutions such as the Internet of Things (IoT), artificial intelligence (AI), and big data analytics further strengthens the potential of biosensor-driven food quality assessment systems. The connectivity provided by the IoT enhances the functionality of sensors, enabling remote monitoring and real-time data collection. Moreover, harnessing AI methods and tapping into the power of big data analytics aids in the identification and analysis of potential issues as well as emerging trends within food samples [71]. Overall, these technological advancements contribute to significantly enhancing the efficiency and intelligence of biosensor-driven food quality assessment systems, ensuring the maintenance of excellent food quality and safety standards.

The fusion of biosensors and artificial intelligence methods in food quality assessment offers multiple advantages. Firstly, this technology achieves high-precision detection by utilizing biosensors for real-time monitoring of trace elements and harmful substances in food, coupled with AI for data processing and analysis. Secondly, the combination of biosensors and artificial intelligence enables rapid detection through real-time data collection, efficient data processing, and analysis, thereby quickly generating assessment results and promptly addressing risks and issues. Additionally, AI methods can predict and provide alerts for potential food quality issues by analyzing extensive data and historical information, thereby identifying abnormal patterns and potential risks. Moreover, the integration of biosensors and artificial intelligence enables real-time monitoring and remote access. Biosensors can collect and transmit data at any time, connected to the cloud via the Internet of Things, while AI algorithms enable remote monitoring, timely handling of anomalies, and providing remote support and solutions. In summary, the fusion of biosensors and artificial intelligence methods brings significant advantages to food quality assessment, including high-precision detection, rapid detection speed, predictive capabilities, real-time monitoring, and remote access. These advantages play a crucial role in ensuring food safety and safeguarding consumer health.

## 5. Conclusions

This paper comprehensively explores the synergistic integration of artificial intelligence and biosensors in food quality assessment, emphasizing their advantages and limitations. Through in-depth analysis, we highlight the importance of multi-modal feature fusion for an innovative food quality perception approach. This approach combines unique features extracted by biosensors with advanced artificial intelligence techniques for subsequent processing. Based on the research analysis in this paper, we believe that researchers in the field of food quality assessment can tailor their methods according to the specific food categories they study. By fully leveraging multiple biosensors and effectively integrating bioelectric signals with image features, researchers can significantly enhance the comprehensiveness and accuracy of food quality perception and classification, thereby improving the overall assessment process. This integrated approach not only helps address the inherent limitations of single-modal perception and human dependence but also provides important reference and inspiration for future research in the field of food quality assessment.

## Data Availability

No new data were created or analyzed in this study. Data sharing is not applicable to this article.
